# Supplementary material for: Sustained-input switches for transcription factors and microRNAs are central building blocks of eukaryotic gene circuits
Source: Genome Biol. 2013 Aug 23;14(8):R85. doi: 10.1186/gb-2013-14-8-r85 (PMC4054853; doi:10.1186/gb-2013-14-8-r85)
Supplement: Additional file 5 — HTML Browsable Motif Output. Zipped folder containing all WaRSwap and FANMOD motif output, viewable in a web browser. [file gb-2013-14-8-r85-S5.ZIP › HTML_browsable_motif_output/WaRSwap_all_tair9/sigs_WSWRSLDraw-2000_all.pvals.heatmaps.html/motif_id_46_000101110_tftype_all_upstream_-3000_0.html]

```
BG_MODEL = Rnew2
MOTIF_ID = 46_000101110
TF_TYPE = all
UPSTREAM = -3000_0


PVals
FN_0.2	FN_0.4	FN_0.6	FN_0.8
dg_60.genes	0.015	0.004	0.07	0.63
dg_70.genes	0.009	0.007	0.066	0.63
dg_80.genes	0.009	0.004	0.069	0.657

ZScores
FN_0.2	FN_0.4	FN_0.6	FN_0.8
dg_60.genes	2.235	2.381	1.475	-0.428
dg_70.genes	2.315	2.49	1.464	-0.436
dg_80.genes	2.256	2.419	1.443	-0.49

StDevs
FN_0.2	FN_0.4	FN_0.6	FN_0.8
dg_60.genes	2847.496	1023.131	309.892	23.741
dg_70.genes	2725.294	1002.562	309.815	24.053
dg_80.genes	2827.905	1015.332	313.893	23.787
```
